# Analyzing Global Components in Developmental Dyscalculia and Dyslexia

**DOI:** 10.3389/fpsyg.2018.00171

**Published:** 2018-02-20

**Authors:** Gloria Di Filippo, Pierluigi Zoccolotti

**Affiliations:** ^1^Faculty of Educational Sciences, Niccolò Cusano University, Rome, Italy; ^2^Department of Psychology, Sapienza University of Rome, Rome, Italy; ^3^Neuropsychological Unit, IRCCS Santa Lucia Foundation, Rome, Italy

**Keywords:** dyscalculia, dyslexia, co-morbidity, reading, numerical cognition

## Abstract

The study examined whether developmental deficits in reading and numerical skills could be expressed in terms of global factors by reference to the rate and amount (RAM) and difference engine (DEM) models. From a sample of 325 fifth grade children, we identified 5 children with dyslexia, 16 with dyscalculia, 7 with a “mixed pattern,” and 49 control children. Children were asked to read aloud words presented individually that varied for frequency and length and to respond (either vocally or manually) to a series of simple number tasks (addition, subtraction, number reading, and number comparisons). Reaction times were measured. Results indicated that the deficit of children with dyscalculia and children with a mixed pattern on numerical tasks could be explained by a single global factor, similarly to the reading deficit shown by children with dyslexia. As predicted by the DEM, increases in task difficulty were accompanied by a corresponding increase in inter-individual variability for both the reading and numerical tasks. These relationships were constant across the four groups of children but differed in terms of slope and intercept on the *x*-axis, indicating that two different general rules underlie performance in reading and numerical skills. The study shows for the first time that, as previously shown for reading, also numerical performance can be explained with reference to a global factor. The advantage of this approach is that it takes into account the over-additivity effect, i.e., the presence of larger group differences in the case of more difficult conditions over and above the characteristics of the experimental conditions. It is concluded that reference to models such as the RAM and DEM can be useful in delineating the characteristics of the dyscalculic deficit as well as in the description of co-morbid disturbances, as in the case of dyslexia and dyscalculia.

## Introduction

In previous research on developmental dyslexia we showed the efficacy of examining performance across reading conditions with reference to a global factor (Zoccolotti et al., 2008). Here we describe a study in which the same approach was extended to the study of developmental dyscalculia.

According to the rate and amount model (hereafter RAM, Faust et al., 1999), group differences in speeded tasks are explained by a multiplicative interaction between a factor that marks the difficulty in any given condition (“*amount*”) and one that marks the global slowness of a group across conditions (“*rate*”). Describing group differences in terms of a global factor allows controlling for the presence of over-additivity, i.e., the tendency of more difficult conditions to yield larger group differences over and above the influence of specific experimental manipulations. Indeed, this is typical of results obtained in various conditions such as Alzheimer’s disease (Myerson et al., 1998) and traumatic brain injury (Ferraro, 1996).

The largest group of studies concerns the effect of aging (e.g., Verhaeghen and Cerella, 2002). When compared to younger adults, older individuals show group differences that are progressively larger in more difficult conditions; this effect can be expressed by contrasting in the same plot (often referred to as the Brinley plot) the condition means of the slower and the faster group (Cerella, 1985). The data points lie on a single regression line and the slope of this regression provides some information on the degree of overall impairment of the slower group. According to the RAM (Faust et al., 1999), the slowness shown by older people indicates a rate factor which interacts multiplicatively with the difficulty of the tasks over and above the specific characteristics of the target conditions.

In various studies, we applied this approach to the study of dyslexia (Zoccolotti et al., 2008; De Luca et al., 2010; Paizi et al., 2013; Martelli et al., 2014). By controlling for the influence of over-additivity, it is possible to examine which factors (if any) are genuinely involved in the reading deficit and which group differences can be parsimoniously interpreted as due to over-additivity. We found that the impairment of children with dyslexia concerns all tasks requiring the processing of strings of letters, such as word and pseudo-word reading or lexical decision tasks, and that this deficit can be almost entirely explained by a single global factor which interacts multiplicatively with the difficulty of the experimental conditions (Zoccolotti et al., 2008; Paizi et al., 2013). Thus, children with dyslexia show greater impairment with pseudo-words than words than typically developing children, but this greater lexicality effect can be entirely explained in terms of over-additivity; a similar pattern is present in the case of word frequency (Zoccolotti et al., 2008; Paizi et al., 2013). By contrast, a residual specific influence of length was found in some (though not all) studies: children with dyslexia are selectively impaired in the case of long words even after controlling for over-additivity (Zoccolotti et al., 2008).

Research on dyslexia has also evidenced a number of conditions in which performance of children with dyslexia is entirely (or largely) spared. Thus, children with dyslexia are impaired when they read words but not when they name the corresponding pictures (Zoccolotti et al., 2008; De Luca et al., 2017). Furthermore, they perform slowly when word and pseudo-word reading and lexical decision tasks are presented in the visual modality but not when they are asked to repeat or make lexical decisions about the same words presented in the auditory modality (Marinelli et al., 2011). Finally, children with dyslexia are impaired in the case of strings of letters (words and non-words) but not (or minimally) when the stimulus is either a single letter or a bigram (De Luca et al., 2010).

These findings raise the question about how to define the scope of the global factor that accounts for the reading deficit in dyslexia. Based on previous results, it appears that the key deficit concerns the processing of visually presented letter strings independent of their lexical value. A similar proposal was put forward by Marsh and Hillis (2005) based on data from neuroimaging studies and from patients with acquired reading deficits. The authors proposed the key role of the “grapheme description,” a pre-lexical orthographic computation independent of case, font, location or orientation. A similar model was proposed by Dehaene et al. (2005) on the basis of their imaging studies on the so-called Visual Word Form Area (VWFA); at both a neural and behavioral level, the Local Combination Detector model envisages the various stages of visual processing which make it possible to process orthographic strings regardless of their location, font and size.

One model designed by Myerson et al. (2003) to formally describe the characteristics of the global factor underlying the differences between groups varying for overall speed of processing is the “*difference engine model*” or DEM. While the RAM focuses on evaluating the presence of specific factors once the over-additivity effect is controlled for, the DEM aims to describe the characteristics of the global factor itself. Therefore, the two models provide complementary information on the role of global and specific components in performance (Myerson et al., 2003); thus, we will refer to both models in the present study.

According to the DEM, the presence of a global factor is defined by the presence of co-variance between difficulty level (as defined by the condition means) and inter-individual variability (as assessed by the corresponding standard deviations, SD). Thus, with increasing difficulty (i.e., slower reaction times, RTs) SDs tend to grow systematically. This largely linear trend has a negative intercept on the *y*-axis and, thus, a positive intercept on the *x*-axis. Myerson et al. (2003) propose that performance on timed tasks can be ascribed to two different (and independent) components (named “*compartments*”) that account for the overall response, i.e., a decisional compartment and a sensory-motor compartment. The intercept on the *x*-axis represents an estimate of the duration of the sensory-motor processing (sensory-motor compartment), which is expected to be constant across a large variety of tasks that require the planning of a minimal motor response. The cognitive component is marked by the co-variation between condition means and SDs. According to Myerson et al. (2003), the slope of the regression between condition means and SDs indicates the degree of correlation among the durations of the processing steps involved in the performances. In this vein, some individuals tend to have brief processing steps while others tend to have longer processing steps. It must be observed that the analysis made by DEM regards the steps in general terms while it does not consider the specific characteristic of the processing stages. In this perspective, the regression between condition means and SDs is the basic relationship which is hypothesized to hold independent of condition difficulty and group differences in speed of information processing. In the same vein, Wagenmakers and Brown (2007) consider the linear relationship between the mean and the SD of a response time distribution as a general law for RT responses under time pressure in decisional tasks.

The DEM specifies the general rules underlying global differences in performance between groups with different basic levels of information processing but does not specify the expected values of the critical parameters (i.e., the time of the sensory-motor compartment and the slope of the regression between means and SDs). In fact, the latter are empirically defined. Based on the analysis of a large set of conditions, Myerson et al. (2003) noted that most cognitive tasks can be described by a relationship between means and SDs with a slope of ca. 30 and a *x*-intercept of ca. 300 ms.

Focusing on global changes in performance does not mean that all cognitive performances are equally impaired in a given group. For example, it has been reported that older individuals are more impaired in visual-spatial than verbal, lexical tasks (Hale and Myerson, 1996; Lawrence et al., 1998). According to the DEM these impairments indicate deficits in selective “domains,” i.e., “verbal-lexical” or “visuo-spatial” domains. Deficits are global because within a given domain the impairment is predicated by a single regression in a Brinley plot; i.e., performances on verbal tasks are explained by a single multiplicative factor and the same holds true for visuo-spatial tasks. At the same time, they are distinct as slopes are clearly different for verbal and visuo-spatial tasks (Lawrence et al., 1998). Even though older individuals are differently impaired on verbal and non-verbal tasks this does not detract from the general law governing the relationship between mean RTs and SDs. Thus, the verbal and non-verbal tasks of both older and younger groups share the same relationship between condition means and SDs (see Figure 14, Myerson et al., 2003). Myerson et al. (2003) made a clear distinction between the general law defining the global factor, which holds across tasks and groups of individuals with different cognitive speeds, and the presence of domain selective deficits, i.e., the observation that a given group of individuals may be impaired on only a sub-set of tasks.

According to the DEM different domains can be accommodated within the same general law of processing. However, this does not necessarily imply that a single, general rule accounts for all possible tasks. In fact, the possibility should be considered that different general relationships hold for different sets of tasks. Notably, neither the RAM nor the DEM take this possibility into consideration but neither do they explicitly rule it out. Both models were devised to examine the possibility of accounting for several group differences using a limited set of predictors; so, their general aim was in this direction. However, it is not incompatible with these models that different general rules apply to different sets of tasks. Empirically, we have noted that the relationship between means and SDs in the case of reading words and pseudo-words was appreciably higher (0.70) than that observed in the case of tasks on single letters and bigrams (0.40; De Luca et al., 2010). Based on these observations we re-analyzed a large dataset from previous experiments on children with dyslexia and control children comparing performances in tasks of lexical decision and reading aloud. In the case of lexical decision tasks, the parameters were similar to previously reported values (i.e., slope of about 0.30 and *x*-intercept of about 300 ms; Zoccolotti et al., 2017). In the case of reading, the slope was considerably steeper (0.66) and the intercept longer (482.6 ms). Therefore, in the case of reading the inter-individual variability is very sensitive to the level of difficulty of the conditions such that even small increases in conditions difficulty produce large increases in inter-individual variability. By contrast, performances in lexical decision tasks indicate parameters similar to several other tasks reported in the literature. Overall, it appears that the general rule underlying the reading task may, indeed, be different from that of most other tasks used in the literature. Notably, these involve a relatively limited number of response alternatives; by contrast, reading requires the identification of a target among many alternatives (in fact, thousands of alternatives). We tentatively proposed that the requirement for a close coupling between orthographic and phonological processing, characteristic of reading tasks, drives the particularly high relationship between performance and inter-individual variability (Zoccolotti et al., 2017). In this view, a peculiar characteristic of reading is that seemingly small increases in difficulty can produce a large increase in variability, i.e., great changes in the tails of the distribution.

In the present research, we applied the same approach to the study of deficits in number processing and calculation. One line of research in dyscalculia has worked on the idea that the disturbance can be interpreted in terms of a deficient numerical processing system or approximate number system (ANS). In turn, this hypothesis rests on the evidence of a “number module” (Landerl et al., 2004; Butterworth, 2005; see also Wilson and Dehaene, 2007). The idea that a deficit in numerical processing is the core deficit in developmental dyscalculia is an interesting working hypothesis from the standpoint of searching for global components in the disturbance.

However, it should be noted that several different theoretical proposals have been put forward in the last years. According to Noël and Rousselle (2011), the deficit in the ANS is actually originated by a developmentally earlier disturbance in the ability to build an exact representation of numerical values. Evidence in favor of this hypothesis comes from the observation that deficits in tasks requiring the comparison of non-symbolic numbers are not present in younger children with dyscalculia (e.g., de Smedt and Gilmore, 2011) though they may emerge later in development (Mazzocco et al., 2011). Other authors have emphasized the heterogeneity of the dyscalculic deficit (Fias et al., 2013; Noël et al., 2016) and have proposed that a number of factors may contribute in generating the developmental deficit. For example, recent evidence points to the idea that short-term visuo-spatial memory and inhibition may be critical in distinguishing between children with and without numerical deficits after controlling for several factors including age, IQ etc. (Szucs et al., 2013). On the other hand, it has been observed that the idea that cognitive factors may modulate the performance in numerical tasks and affect the emergence of the deficit does not necessarily detract from the hypothesis that the core deficit of the disturbance rests upon a deficient numerical processing system (Landerl et al., 2013).

To distinguish between reading and calculation, we examined children with deficits in one (or both) of these areas. It is well known that dyslexia and dyscalculia can appear in isolated forms (Rubinsten and Henik, 2006) but tend to be co-morbid (e.g., Lewis et al., 1994; Badian, 1999; Barbaresi et al., 2005; Tressoldi et al., 2007; Dirks et al., 2008). Recent studies investigated the nature of the different cognitive correlates of this co-morbidity (Willburger et al., 2008; Landerl et al., 2009; Wilson et al., 2015). Landerl et al. (2009) propose that a deficit in phonological processing is critical in the case of dyslexia while one in a number module is critical in dyscalculia. Partially similar results were reported in the case of young adults with dyscalculia (Wilson et al., 2015). In line with the co-morbidity perspective (Pennington, 2006), cognitive deficits in the comorbid dyslexia/dyscalculia group could be accounted for by a combination of the two learning disorders, i.e., the effects were additive (Landerl et al., 2009). Recent research has also tried to detect deficits that could account for the shared variance in the two disorders, but results are still variable. Wilson et al. (2015) reported that candidates, such as domain general deficits in rapid naming or in verbal short-term memory, did not explain the co-morbidity (Wilson et al., 2015). By contrast, Slot et al. (2016) reported that the shared risk for reading and calculation could be accounted for in terms of weakness in phonological awareness tasks.

In the present study, we set out to verify whether disturbances in reading and numerical tasks could be described in terms global factors in both isolated and co-morbid cases. Our general expectation was that children with dyslexia would be delayed in reading tasks and that their performances across conditions would be accounted for by a single regression (with a relatively steep slope) across all reading tasks but not (or minimally) numerical tasks. We expected the opposite in the case of children with dyscalculia, i.e., that their performance across numerical tasks would be accounted for by a single regression line and their performance on reading tasks would be minimally affected. Finally, we expected that co-morbid cases would perform pathologically on both sets of tasks.

With regard to the DEM, one interesting question is whether the putatively differential deficits in reading and numerical tasks can be explained in terms of different “domains” or, alternatively, in terms of different general laws of performance. In the former case, one would expect children with dyslexia or dyscalculia to be impaired only in the reading or numerical domain, but these two domains would show the same relationship between RT condition means and SDs. In the latter case, one would expect that reading and numerical tasks would actually show distinct relationships between means and SDs. Based on our recent re-analysis (Zoccolotti et al., 2017), the relationship between means and SDs can be hypothesized as steeper in the case of reading tasks than numerical tasks.

Overall, the aims of the study were to the following:

- To ascertain whether performance on numerical tasks can be effectively described in terms of a global factor putatively referring to the efficiency of a number module;
- To establish whether the expected difference between numerical and reading tasks can be most effectively described in terms of different domains and/or in terms of different underlying rules governing the relationship between means and SDs;
- To determine whether the co-morbidity between reading and numerical tasks can be adequately described in terms of global factors.

## Materials and Methods

### Sample

Children were examined in five different schools in Rome in a middle-class environment. All the 22 classes in these schools participated in the study. Approximately 15 children per class accepted to participate in the study (out of an average of ca. 20 per class). Overall the sample included a total of 325 fifth graders (178 Male and 147 Female; mean age = 10.6).

Identification of reading and calculation deficits was based on the standards identified by the Consensus Conference on specific learning disabilities (Istituto Superiore di Sanità, 2011). To detect calculation deficits, it is required to use tasks involving specific skills (such as arithmetic facts, additions, subtractions, number comparisons etc.). The performance of the child should be expressed in terms of standard deviations from a normative sample (not in terms of age equivalents). Both tasks measuring accuracy and speed are envisaged. To detect reading deficits, it is required that children show a deficit in both accuracy and speed based on a quantitative comparison with standard norms. Performance on reading comprehension is not diagnostic of the reading deficit although it provides complementary information for the functional framing of the disturbance. It is envisaged that lists of words and non-words are included in the test battery as they provide particularly effective measure of reading deficit.

Children were initially administered two standard tests to detect reading and calculation deficits. As for calculation skills, the group-administered part of AC-MT test of (Cornoldi et al., 2002) includes subtests that investigate addition and subtraction, multiplication and division, number size comparisons, digit transformation, and number ordering. Two overall scores are derived, one for Written operations and one for Numerical knowledge. Normative values for these two scores are available from Cornoldi et al. (2002). As for reading, the MT test for elementary school (Cornoldi and Colpo, 1998) was individually administered to the children. In the MT reading test, the child reads aloud a text passage with a 4-min time limit; speed (s per syllable) and accuracy (number of errors, adjusted for the amount of text read) are scored and can be expressed as *z* scores with reference to normative values (Cornoldi and Colpo, 1998). The MT scale also envisages a comprehension sub-test; following clinical standards (Istituto Superiore di Sanità, 2011) this test was not used as part of the selection criteria. In this case, the child reads a second passage silently, with no time limit, and responds to 10 multiple-choice questions. The children were also given Raven’s Colored Progressive Matrices to evaluate non-verbal intelligence; only children who performed within normal limits (>10 percentile) were included in the experimental samples.

Based on standard values, we identified children with either a selective deficit in reading, calculation or both. We adopted a *z* score cut-off of -1.65 (corresponding to ca. 5% of children under normality assumptions). Out of the total of 325 children examined, 12 failed in reading accuracy and/or speed (i.e., 3.69%) while 23 children (7.08%) failed either in the Written operations or Numeric knowledge indexes on the AC-MT test (for comments on these proportions see section “Discussion”). Overall, five children (*F* = 4, *M* = 1) only failed in reading and were considered “dyslexic”; 16 children (*F* = 6, *M* = 10) only failed in number tasks and were considered “dyscalculic”; finally, 7 children (*M* = 4, *F* = 3) showed a mixed pattern (i.e., they failed at least one reading and one numerical test), consistent with the high co-morbidity between the two deficits. A control group of 49 children (*F* = 23, *M* = 26) with spared performance on all screening tests was also examined; the children were selected from the overall original sample based on their willingness to participate in the study (but with no further selection based on screening measures).

Performance of the different groups of children on the two screening tests is presented in **Table 1** in terms of mean *z* scores (and SDs) based on normative values. For the AC-MT test of calculation skills, values on the Written operations and Numeric knowledge indexes are presented. For the MT test, data for accuracy and time in reading a text passage are separately presented. Data on comprehension are also shown; note that they indicate only very mild deficiency in children with dyslexia and children with mixed pattern. This is in keeping with previous data on Italian children which indicate that comprehension is only mildly (or minimally) affected (Judica et al., 2002) if the comprehension test does not stress decoding skills (in the case of the MT test, no time limit is given to the child to read the text passage before responding to the multiple-choice questions).

**Table 1:** Performance of the different groups of children in the two screening tests and in the additional reading and number tasks in terms of mean *z* scores (and SDs) based on normative values.

	Controls		Children with dyslexia		Children with dyscalculia		Children with mixed pattern	
	Mean	*SD*	Mean	*SD*	Mean	*SD*	Mean	*SD*
**Reading tasks**								
MT (acc.)	0.01	0.44	-1.90	0.81	-0.70	0.43	-2.24	0.63
MT (time)	0.18	0.45	-3.34	2.65	-0.52	0.71	-3.65	1.87
MT (comprehension)	0.79	0.45	-0.50	1.03	-0.18	1.23	-0.91	1.37
**Reading tasks (additional tests)**								
Short HFW (time)	0.17	0.86	-3.87	3.87	-0.61	1.29	-4.22	3.91
Long HFW (time)	0.31	0.52	-2.74	2.81	-0.27	0.84	-4.39	3.36
Short LFW (time)	0.32	0.56	-4.36	3.91	-0.32	0.77	-3.25	1.46
Long LFW (time)	0.52	0.49	-3.30	3.00	-0.12	0.74	-3.03	1.49
Short NW (time)	0.34	0.56	-2.29	2.54	-0.22	1.07	-3.08	1.01
Long NW (time)	0.31	0.66	-3.71	3.76	-0.50	1.12	-3.13	1.99
Short HFW (acc.)	0.10	0.76	-1.00	1.40	-0.21	1.03	-1.90	2.77
Long HFW (acc.)	-0.28	1.06	-1.78	2.08	-0.46	1.27	-5.65	4.65
Short LFW (acc.)	0.17	0.91	-0.92	1.45	-0.51	1.13	-1.92	2.00
Long LFW (acc.)	0.03	0.81	-1.81	1.56	-0.60	1.09	-2.50	1.54
Short NW (acc.)	0.10	1.16	-2.46	2.34	-0.44	1.16	-2.27	1.95
Long NW (acc.)	-0.09	0.97	-1.85	0.99	-0.69	0.93	-1.36	0.92
**Number tasks (screening)**								
Written operations	0.41	0.68	0.50	0.30	-1.64	0.77	-2.51	0.93
Numeric knowledge	0.39	0.55	-0.76	0.74	-1.27	1.18	-1.63	1.54
**Number tasks (additional tests)**								
Mental calculations	0.05	1.00	-1.04	0.84	-1.10	1.46	-2.85	1.93
Triplets (acc.)	0.45	1.06	-0.14	2.01	-1.23	1.72	-2.79	4.68
Triplets (time)	0.07	0.80	-0.96	0.79	-1.79	2.04	-1.52	1.41
Insertions (acc.)	0.32	0.59	0.31	0.83	0.10	0.95	-1.43	3.32
Insertions (time)	0.25	0.77	-0.57	1.20	-1.00	1.43	-2.64	3.68

A number of other tests were given to confirm the diagnosis on both reading and calculation skills. With regard to reading, we administered the Word and Pseudo-word Reading Test (Zoccolotti et al., 2005). The test includes four lists of 30 words (varying for frequency and length) and two lists of 30 non-words (varying for length). Number of errors and reading times are scored. Mean *z* score data (and SDs) for these lists are reported in **Table 1**. The children with dyslexia and those in the mixed pattern group were severely affected, the mean standardized score being less than two SDs in several conditions. In particular, the children with dyslexia were more affected in time than in accuracy measures; the disturbance was severe in both time and accuracy measures in the children in the mixed pattern group. Word and non-word reading was impaired to a similar extent. Thus, no selective deficit in reading non-words was detected, similarly to previous observations in Italian children (Zoccolotti et al., 1999). As a group, the children with dyscalculia were minimally affected across all measures.

As for numerical tasks, three sub-tests from the Battery for Developmental Dyscalculia (Biancardi and Nicoletti, 2004) were given: In Complex oral calculation sub-test, the child must perform 10 additions and 10 subtractions with results above 10; a time limit of 15 s is given. In triplets, the child must choose the largest number in a set of three Arabic numbers (1–6 digits); twenty trials are given; both accuracy and speed are measured. In the Insertions sub-test, the child must place a 1- to 5-number among three other numbers; 12 trials are given; both accuracy and speed are measured. Mean *z* data (and SDs) for these sub-tests are reported in **Table 1**. Children with dyscalculia were moderately affected in all conditions with the exception of accuracy in the Insertions sub-test. Children in the mixed pattern group were generally more affected across conditions, including accuracy on the Insertions sub-test. As a group, the children with dyslexia were generally spared in most conditions; however, a moderate deficit was detected in the Mental Calculation and in the Triplets (time) sub-tests.

**FIGURE 1:**
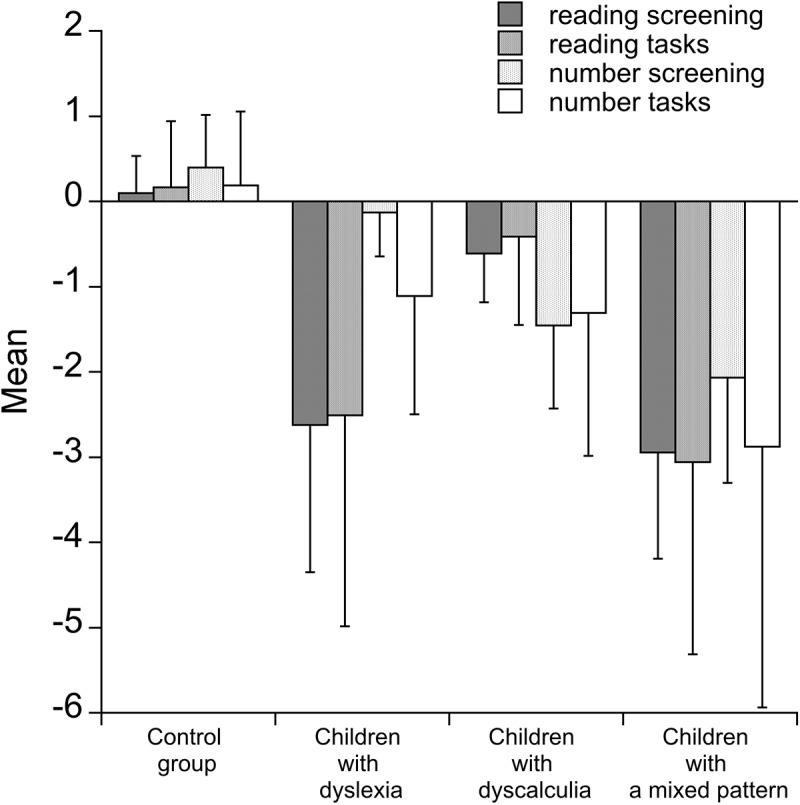
Mean and *z*-score performance (and SDs) in reading and number tasks of the four groups of children (controls, children with dyslexia, children with dyscalculia, and children with a mixed pattern). Values indicate average performance across tests used for screening and for the additional tests used for the evaluation of children (see text for details).

Information about the reading and numerical conditions is summarized in **Figure 1**, where data from different measures of reading and number processing are collapsed into overall indexes separately for the screening and additional measures. Inspection of the figure indicates a somewhat more clear-cut separation between reading and numerical tasks in children with dyslexia than in children with dyscalculia. Children in the mixed pattern group showed a severe deficiency across the two sets of tasks.

Due to the selection criteria, the children generally performed well on Raven’s Progressive Matrices; in particular, the control children’ mean performance was 30.26 (*SD* = 3.35), the performance of children with dyslexia was 26.75 (*SD* = 5.42), the performance of children with dyscalculia was 29.05 (*SD* = 3.96) and the performance of children in the mixed pattern group was 27.29 (*SD* = 5.44). According to Pruneti et al. (1996), normative values indicate a mean performance of 30.2 (*SD* = 4.3) in fifth grade. Therefore, although the performance of children with dyslexia and children in the mixed pattern group was in the normal range it was somewhat lower than expected. However, group differences between the four groups did not reach statistical significance [*F*_(3,79)_ = 2.2, *p* = 0.09].

The study was carried out according to the principles of the 2012–2013 Helsinki Declaration. Written informed consent to participate in the study was obtained from the parents of all children. The study was approved by the IRB of the Department of Psychology of Sapienza University of Rome.

### Experimental Tests

Several experimental tests were given.

To test reading, the children had to read aloud words individually presented at the center of a PC screen. The list of words was derived from Paizi et al. (2013; experiment 3): high- and low-frequency words (based on child-printed frequency counts; Marconi et al., 1993) that varied in letter length from four to seven letters (that varied in length from four to seven letters) for a total of eight different conditions were selected from the LEXVAR database (Barca et al., 2002). There were 15 stimuli in each orthogonal condition for a total of 120 stimuli. The stimuli were presented in five blocks of 24 words each. At the beginning, a practice block was administered; it consisted of 10 words that were different from the experimental items but had the same characteristics. A short pause was allowed after each block.

Bigram frequency was matched across conditions. Initial phonemes in the four sets were matched for the manner of articulation as well as for the voiced vs. voiceless features. N-size, age of acquisition and orthographic complexity were matched between corresponding length sets in the high- and low-frequency conditions. For a full description of the list please refer to Paizi et al. (2013). Vocal reaction times (RT) were measured. Median RTs for each condition was the dependent measure.

For numerical tasks, five sub-tests were used: (1) One-number addition: the child saw a pair of numbers at the center of the PC screen with the + sign in the middle and had to say aloud the result of the addition ASAP (vocal RTs were measured); 20 trials were given; (2) One-number subtraction: similar to the previous sub-test except that the child had to say the product of the subtraction; (3) One-digit number reading: a digit was presented at the center of the PC screen and the child had to read it aloud; twenty trials were given; (4) Two-digit number reading: the same as 3 but the numbers had two digits. (5) Number comparison: two numbers were presented, one on the left and one on the right of the PC screen. The child had to press one of two keys that indicated the highest number. Median RTs were used as the dependent measure.

### Procedure

The screening procedures included both group and individually administered tests, which were given to the children in a single session in a quiet room in their school.

The children who participated in the rest of the study took the additional reading and numerical tests during another individual session.

In the final session, the children were administered the experimental tests. The order of presentation of the reading and calculation tests was counter-balanced across subjects.

### Data Analysis

The RAM and DEM models make predictions in the case of open-scale measures, such as RTs, but not accuracy. Thus, statistical analyses were carried out on RTs while accuracy values were inspected to exclude the possible presence of speed accuracy trade-off data. No such trade-off was detected and accuracy data were not further analyzed.

The RAM and DEM envisage different conditions to identify the presence of a global factor:

(1) The RAM (Faust et al., 1999) predicts a linear relationship between the condition means of two groups varying in terms of overall information-processing rate. Thus, we separately plotted the mean RTs for each group of children with learning problems (i.e., with dyslexia, dyscalculia and with a mixed pattern) against the performance of control children. We expected the group differences to increase linearly with the difficulty of the condition with separate regression lines for reading and numerical tasks. We expected that the children with dyslexia would have a steeper slope for reading than for numerical tasks and that those with dyscalculia would show the opposite pattern.

(2) The DEM (Myerson et al., 2003) predicts a linear relationship between the group means and the corresponding standard deviations. Note that homogeneity of variance is a basic assumption in parametric analyses; however, the presence of a clear-cut linear increase in SDs as a function of condition difficulty marks a systematic deviation from such assumption. To test the model prediction, mean RTs of the different groups of children in the different experimental conditions were plotted as a function of the corresponding SDs; data from the various groups were plotted in the same graph as the DEM predicts that the slope of the regression is constant across different groups of children. Furthermore, the model states that the *x*-intercept represents an estimate of the time for early visual processing, response selection and execution (sensory-motor compartment). Again, the DEM predicts that the same intercept on the *x*-axis would hold across different groups of children. As indicated in the Introduction, recent evidence (Zoccolotti et al., 2017) raises the question about whether reading tasks have the same general parameters as other cognitive timed tasks. To check this possibility, different plots between SDs and means were made for reading and number conditions.

To remove the effect of over-additivity in the data we made analyses on *z*-score data. Following Faust et al. (1999) they are calculated by subtracting the mean of each condition from the overall participant mean and dividing the product by the standard deviation of the condition means for each child; thus, z-transformed values represent the deviation of each condition from the overall participant mean. In this way, global components are controlled for, but individual variability across experimental conditions is preserved. We carried out separate ANOVAs on the reading and numerical data assessing the effects of the different variables marking the conditions of these tasks on both raw and z-transformed RTs. Based on Faust et al. (1999) interactions with the group factor which are significant in the z-transformed data (i.e., controlling for global components) highlight a genuine effect; interactions with the group factor which are significant in the raw, but not in the z-transformed, analyses indicate the presence of over-additivity. Whenever appropriate, means were compared with the Tukey *post hoc* test considering *p* < 0.05 as a reference. The variables entered in the different analyses are spelled out in the Section “Results.”

## Results

### Analysis of Global Factors

**FIGURE 2:**
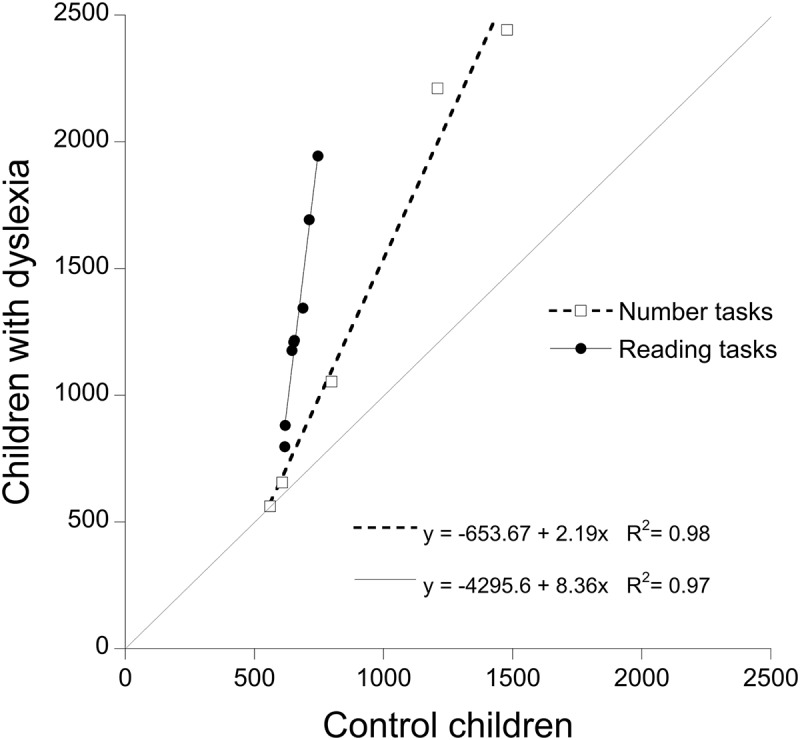
Test of RAM predictions based on results of children with dyslexia and controls: condition means of children with dyslexia are plotted as a function of controls’ means. Open squared report RTs for number tasks; filled circles report RTs for reading tasks. The dotted line (slope = 1) represents the reference for equal performance in the two groups of children.

**Figure 2** presents a Brinley plot to examine the prediction that “*the condition means for a particular group as a function of the condition means for another group will be linear*” (Faust et al., 1999). For children with dyslexia, performance on the word conditions was well fit by a single regression line (*y* = -4295.6 + 8.36x) that explained a large proportion of variance (*R*^2^ = 0.97). Also, the conditions of the numerical tasks were well fit by a single regression line with a different slope (*y* = -653.7 + 2.19x; *R*^2^ = 0.98).

**FIGURE 3:**
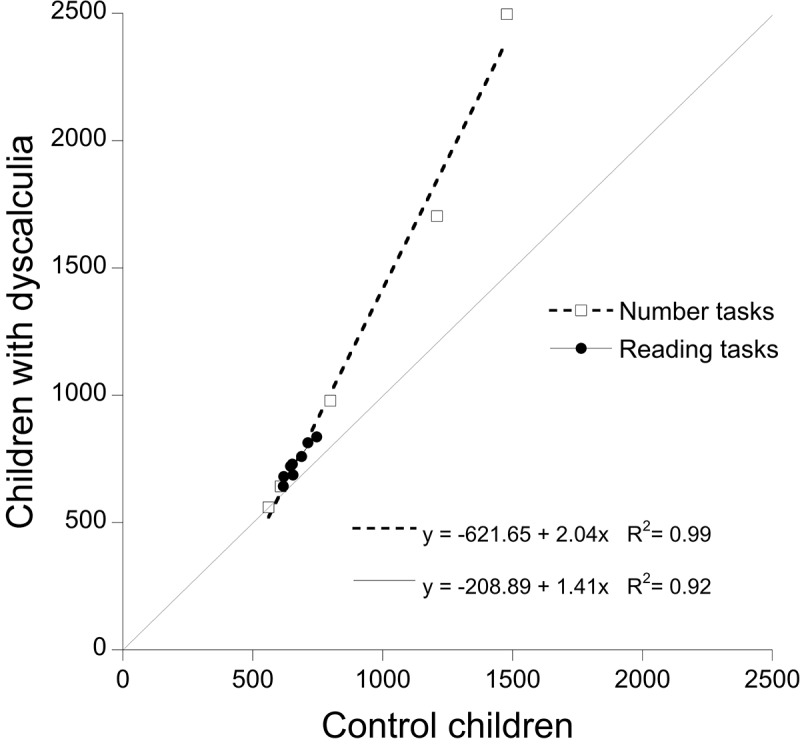
Test of RAM predictions based on results of children with dyscalculia and controls: condition means of children with dyscalculia are plotted as a function of controls’ means. Open squared report RTs for number tasks; filled circles report RTs for reading tasks. The dotted line (slope = 1) represents the reference for equal performance in the two groups of children.

We applied the same approach to children with dyscalculia; the resulting Brinley plot is presented in **Figure 3**. Also in this case, we applied a solution with two regression lines, one for reading (*y* = -208.9 + 1.41x; *R*^2^ = 0.92) and one for numerical (*y* = -621.65 + 2.04x; *R*^2^ = 0.99) tasks, which effectively accounted for the experimental data. Inspection of the figure indicates that children with dyscalculia show a very limited spread of performance in the case of reading tasks (as expected). Furthermore, opposite to children with dyslexia, the slope for calculation tasks was steeper than that for reading tasks although the difference was less marked than in the case of children with dyslexia.

**FIGURE 4:**
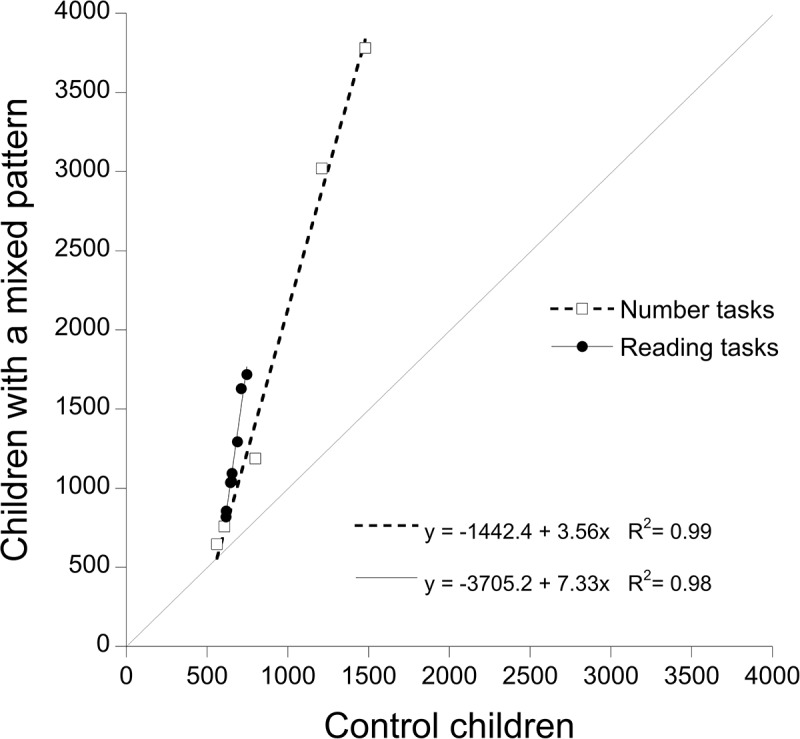
Test of RAM predictions based on results of children in mixed pattern group and controls: condition means of children with a mixed pattern are plotted as a function of controls’ means. Open squared report RTs for number tasks; filled circles report RTs for reading tasks. The dotted line (slope = 1) represents the reference for equal performance in the two groups of children.

The performance of the children with a mixed pattern (dyslexia and dyscalculia) was severely impaired for both reading (*y* = -3705.2 + 7.33x; *R*^2^ = 0.98) and numerical (*y* = -1442.4 + 3.56x; *R*^2^ = 0.99) tasks. The resulting Brinley plot is presented in **Figure 4**. Note that the performance of these children is considerably more impaired; thus, the figure has a much larger scale than the two previous graphs.

**FIGURE 5:**
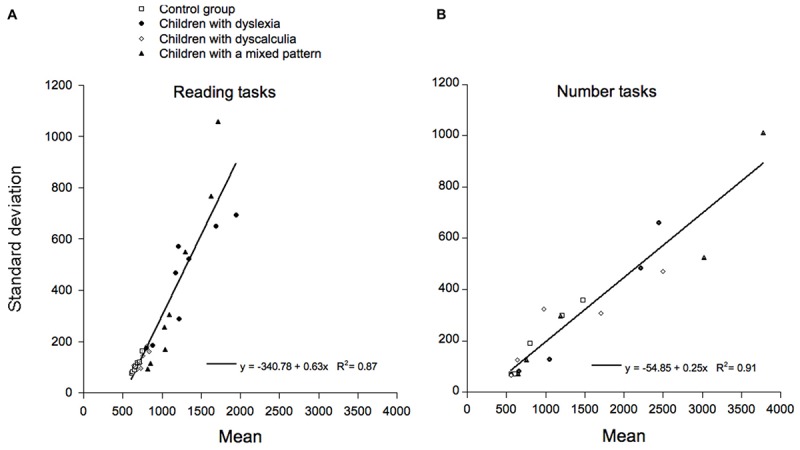
Test of DEM predictions: SDs for each group and condition are reported as a function of the corresponding means. Different symbols refer to data of the four different groups of children (controls, children with dyslexia, children with dyscalculia, and children with a mixed pattern). Data for the reading and for the number tasks are separately reported in **(A,B)** respectively.

We then tested the DEM prediction (Myerson et al., 2003) that there should be a linear relationship between the group means and the standard deviations in the same conditions. Data for the reading and number tasks are presented separately in **Figure 5A** (reading tasks) and **Figure 5B** (number tasks).

A number of general characteristics emerge in these plots. Data in plot 5a indicate that a single regression line accounts quite well for the performance of all four groups of children on the number tasks (with a *R*^2^ = 0.91). The slope of the relationship is 0.25 and the intercept on the *x*-axis is 219.4. As one intercept on the *x*-axis accounts well for the data of all sub-groups of children, based on DEM this indicates that they are not different in the sensory-motor (non-decisional) compartment but only in the cognitive compartment. If separate regression lines are used for the four groups of children, slopes vary between 0.18 and 0.32 and determination coefficients vary between 0.82 and 0.97. As only one numerical sub-test required a manual response, we also re-examined the relationship between means and SDs excluding this sub-test. The results were very similar: the slope of the relationship was 0.25 and the intercept on the *x*-axis was 280.5 (with a *R*^2^ = 0.93).

Also for reading tasks, a single regression line accounts well for the responses of all groups (with a *R*^2^ = 0.87). In this case, the relationship is 0.63 and the intercept on the *x*-axis is 537.25 ms. Again, one intercept on the *x*-axis accounts well for the data of all sub-groups; this is in keeping with the idea that groups are not different in the sensory-motor (non-decisional) compartment but only in the cognitive compartment. If separate regression lines are used for the four groups of children, slopes vary between 0.47 and 1.02 and determination coefficients vary between 0.71 and 0.96.

Overall, our data indicate that the same relationship between mean performance and variability holds for all groups of children, as predicted by DEM. However, data relative to the orthographic and numerical tasks also indicate distinct linear relationships in terms of both slopes and intercepts on the *x*-axis. This pattern is consistent with the idea that reading and numerical tasks do not merely represent two separate domains; in fact, performances in these two sets of data point to the presence of two separate general relationships between means and SDs. Further comments on this point will be presented in the Section “Discussion.”

### Anovas

#### Reading

Two ANOVAs were carried-out on mean RTs and z-transformed values with length (4-, 5-, 6-, and 7-letter words) and frequency (high-low) as repeated measures and group (controls, children with dyslexia, children with dyscalculia and mixed group) as unrepeated measure. The ANOVA on raw RTs showed a main significant effect of the group factor in the raw [*F*_rt(1,79)_ = 34.14, *p* < 0.0001, $\eta_{p}^{2}$ = 0.56], but (due to the data transformation) not in the z-transformed analysis (*F*_z_ < 1, n.s.; *F*_rt_ refers to the raw data analysis and *F*_z_ to the z-transformed analysis). On average control children responded in 666.8 ms, which was significantly faster than the RTs of children with dyslexia (1282.9 ms) and of children in the mixed pattern group (1279.4 ms), who did not differ from each other. The RTs of children with dyscalculia were insignificantly slower than those of controls (734.4 ms) but slower than those with dyslexia and with a mixed pattern. The effect of word frequency [*F*_rt(1,79)_ = 62.18, *p* < 0.0001, $\eta_{p}^{2}$ = 0.44; *F*_z(1,79)_ = 20.70, *p* < 0.0001, $\eta_{p}^{2}$ = 0.63] was significant, indicating faster RTs for high- (732.3 ms) than low-frequency (893.6 ms) words (diff. = 161.3 ms). The main effect of length [*F*_rt(3,237)_ = 96.39, *p* < 0.0001, $\eta_{p}^{2}$ = 0.55; *F*_z(3,237)_ = 102.92, *p* < 0.0001, $\eta_{p}^{2}$ = 0.51] was significant, indicating slower RTs for longer words (with an average 82.9 ms increase per letter). The frequency by length interaction was significant [*F*_rt(3,237)_ = 13.97, *p* < 0.0001, $\eta_{p}^{2}$ = 0.15; *F*_z(3,237)_ = 3.75, *p* = 0.01, $\eta_{p}^{2}$ = 0.05] indicating larger length effects for low- than for high-frequency words. All interactions with the group factor (group by length, group by frequency and group by length by frequency) were significant (at least *p* < 0.01) in the raw data analysis; however, they all vanished in the *z*-score analysis (all *F*_s_ < 1.1, n.s.; all $\eta_{p}^{2}$ < 0.04), indicating that they could all be accounted for in terms of over-additivity.

#### Numerical Tasks

Two ANOVAs were carried-out on raw RTs and z-transformed values with condition (one-digit number reading, two-digit number reading, number comparison, one-number addition and one-number subtraction) as repeated measure and group (controls, children with dyslexia, children with dyscalculia and mixed group) as unrepeated measure. The ANOVA on mean RTs showed a main significant effect of group in the raw data [*F*_rt(3,79)_ = 31.03, *p* < 0.0001, $\eta_{p}^{2}$ = 0.54], but was inherently nil in the z-transformed analysis. On average RTs of control children (931.3 ms) were significantly faster than RTs of children with dyscalculia (1276.2 ms) and of children in the mixed pattern group (2130.7 ms). The RTs of children in the mixed pattern group also differed from those of children with dyslexia and dyscalculia. The difference between controls and children with dyslexia (1384.8) fell short of significance (*p* = 0.08). The effect of condition was significant [*F*_rt(4,316)_ = 88.40, *p* < 0.0001, $\eta_{p}^{2}$ = 0.53; *F*_z(1,91)_ = 634.51, *p* < 0.0001, $\eta_{p}^{2}$ = 0.89]: RTs to one- (575.0 ms) or two-digit (641.9) number reading yielded the shortest (and not significantly different from each other) RTs whereas one-number additions (1668.3 ms) and one-number subtractions (2259.7) were slower (and significantly different from each other); RTs for number comparison (920.0 ms) were intermediate (and significantly different from the one-number addition and subtraction conditions). The group by condition interaction was significant in the raw data analysis [*F*_rt(12,316)_ = 14.87, *p* < 0.0001, $\eta_{p}^{2}$ = 0.36], but vanished in the z-transformed analysis [*F*_z(12,316)_ < 1, n.s.; $\eta_{p}^{2}$ = 0.04], indicating the presence of over-additivity.

## Discussion

The first aim of the present study was to establish whether performance on numerical tasks could be described in terms of a global factor. Results are clearly in favor of this hypothesis. For example, RTs of children with dyscalculia grew by a slope of 2.04 as a function of condition difficulty with respect to the performance of control children and this regression accounted for a very large proportion of variance (*R*^2^ = 0.99). This indicates that differences in raw data between children with dyscalculia and controls increase as a function of condition difficulty over and above the specific characteristics of the experimental conditions, pointing to the presence of an over-additive effect for numerical tasks. Consistently, when analyses were carried out to remove the effect of over-additivity by using z-transformed data, conditions requiring additions, subtractions, number comparisons etc. yielded about the same group differences between dyscalculic children and controls. These results are consistent with the idea that performance on numerical tasks can actually be described in terms of a single global factor. Interestingly, a line of research has focused on the idea that performance on different numerical and calculation tasks can be seen in terms of a number module (Landerl et al., 2004; Butterworth, 2005). The present results are broadly in keeping with this idea. However, it might be necessary to examine a larger variety of numerical tasks before a definite conclusion can be reached on this point. In particular, only symbolic stimuli were used; extension of these results to non-symbolic stimuli is required before a clear statement on the number module hypothesis can be made.

Therefore, a large proportion of the variability across number tasks can be accounted for in terms of global components in the data. Models, such as the RAM and DEM, help to define the characteristics of these components. One intriguing question concerns whether clusters of tasks can be expressed in terms of different domains or in terms of different general rules or laws, governing the relationship between performance and inter-individual variability. An example of a distinction in terms of domains is provided by the studies on aging (Hale and Myerson, 1996; Lawrence et al., 1998) which indicate a greater impairment in visuo-spatial than in verbal tasks, although the general relationship underlying these tasks was the same. However, recent evidence indicates that reading tasks may actually point to the presence of a different general law in the data. In a re-analysis of a large set of previous experiments examining vocal RTs to reading isolated words (Zoccolotti et al., 2017) we noted that the regression between means and SDs had a much steeper slope and a larger *x*-intercept. Accordingly, reading (but not lexical decision) tasks map onto a different general relationship so that inter-individual variability grows at a particularly high rate also with moderate increases in condition difficulty.

In this study, we were able to test this hypothesis on non-retrospective data by comparing performance on reading and numerical tasks. The results clearly support the idea that the general relationship between means and SDs is quite different for reading and numerical tasks. In particular, the slope was considerably higher in reading (0.63) than in numerical (0.25) tasks. Furthermore, a larger intercept on the *x*-axis was present for reading (537.25 ms) than for numerical tasks (280.54 ms). On the one hand, the pattern for the number tasks is quite similar to the parameters reported for several visuo-spatial and verbal tasks by Myerson et al. (2003). On the other hand, the pattern for the reading tasks closely replicates the results reported in our retrospective analysis, where the slope was 0.66 and the intercept on the *x*-axis was 482.6 ms. Therefore, the present data are in keeping with the idea that the differences between reading and numerical tasks do not merely point to the presence of two different domains but actually refer to the presence of two general laws underlying these two sets of data. In trying to understand the origin of this quite general distinction, it is interesting to note that, with one exception (i.e., the number comparison task) all tasks used in the present research required a vocal response. Thus, the present data exclude the possibility that the difference may lie in the nature of the response. Indeed, all tasks used in the studies by Myerson et al. (2003) envisaged a manual response; so, this possibility deserved some consideration. Alternatively, we have proposed that reading is different from all other tasks as it involves a situation in which a very large set of alternative responses is present; indeed, the observer has to name a word from a possible pool of thousands of alternatives. According to the DEM, the slope of the regression between the means and the SDs indicates the degree of correlation among the durations of the processing stages. To identify a target the observer has to closely couple the output of the orthographic analysis with the identification of the corresponding phonological code and we have proposed that it may be this requirement that drives the particularly steep relationship between performance and inter-individual variability in reading tasks (Zoccolotti et al., 2017).

We searched for associated and dissociated reading and number difficulties starting from a moderately large school sample. Evidence indicated that these two sets of deficits frequently co-occurred, as expected. Out of a total of 325 children, 12 showed reading deficits (i.e., 3.69%). This figure is very similar to Barbiero et al.’s (2012) recent Italian prevalence data. These authors reported a proportion of children with dyslexia comprised between 3.1 and 3.2%. More than half of children with a reading deficit (7 or 58.3%) also failed on numerical tasks. Proportion of children with deficits in numerical tasks was higher: 23 children or 7.08%. As yet, no systematic epidemiological data are available in Italian for this deficit; however, it has been recently reported that prevalence estimates range around 6% (e.g., Wilson et al., 2015). Thus, the proportion of children with deficits in numerical tasks in the present study appears in line with the current available data in the literature. Seven of the 23 children with numerical deficits (i.e., 30.4%) also showed reading deficits. Thus, the overlap between the two disturbances was high. This is in keeping with data from the literature; for example, Wilson et al. (2015) estimated that relative overlap between the two disorders averages 37% across different studies. Thus, the present data broadly fit with the figures reported in the literature both in terms of their separate prevalence and in terms of the overlap between the two disorders. Interestingly, although children were in the normal range in intelligence measures there was a tendency for children in the mixed group and in the dyslexic group to score lower than children with dyscalculia or controls in the Raven test. Recent evidence indicates differences in intelligence measures among groups with different learning deficits (Cornoldi et al., 2014; Giofrè et al., 2017; Toffalini et al., 2017). It may be interesting to verify the stability of these differences by using larger groups of children.

From these general figures, we identified two groups of children with putatively isolated reading and numerical deficits and a group with co-morbid symptoms. Empirically, data from both the standard cognitive tests and from the experimental tasks indicated that the dissociation between reading and numerical skills was incomplete in the dyslexic as well as in the dyscalculic group; in other terms, children with dyslexia were not entirely spared in number tasks and vice versa. Different factors may contribute to this outcome. First, dyslexia and dyscalculia are currently considered graded difficulties and cut-offs always maintain a certain degree of arbitrariness (e.g., Pennington and Bishop, 2009). Thus, incomplete dissociation of symptoms may actually be “real” in the sense that co-morbidity in itself may be seen as a graded phenomenon rather than as a categorical one. Alternatively, it is possible that the tests used were only partially sensitive to the underlying dimensions and that some true co-morbid cases were not detected because of the insensitivity of the measures used.

At any rate, the focus of the present research was on the possibility of expressing dissociations and associations between reading and numerical conditions in terms of global components. A partial dissociation between reading and numerical tasks was clear in children with dyslexia in terms of very different slopes in the Brinley plot between the two sets of tasks (8.36 for the reading and 2.19 for the number tasks, respectively). In children with dyscalculia, the pattern was reversed but the difference between the two slopes was much smaller (1.41 for the reading and 2.04 for the number tasks, respectively). In this case (see **Figure 3**) what seems to discriminate best between the two sets of tasks in this group of children is the presence of a very small range of variability across reading conditions. As discussed above, at a global level of analysis, reading is characterized by a very tight relationship between difficulty level and inter-individual variability. In these terms, seemingly small increases in condition difficulty will generate comparatively large group condition differences between affected and unaffected individuals. Comparing the reading means of **Figures 2**, **3** makes it clear that, unlike what happens in children with dyslexia, in those with dyscalculia this “explosion” does not take place and differences among reading conditions amount to tens (rather than hundreds) of milliseconds, i.e., in a range very similar to that of controls. Results of children in the mixed pattern group indicate that the pattern of deficiencies in reading and numerical tasks is well accounted for by two regression lines with large slopes (7.33 for the reading and 3.56 for the number tasks, respectively). Overall, it appears that reference to global components provides a good description of both isolated and co-morbid deficiencies. Notably, however, the two sets of data do not yield specular profiles. This is probably due to the fact that different general laws underlie the two sets of tasks. In particular, the presence of a very tight relationship between condition means and inter-individual variability (which is characteristic of reading) is expressed very clearly producing a large spread of performances across different conditions in the affected children (those with dyslexia and with a mixed pattern) but not in the unaffected ones (i.e., children with dyscalculia).

Although research on co-morbidity has increased considerably in recent years, partly due to the seminal work of Pennington (e.g., Pennington, 2006; Pennington and Bishop, 2009), full comprehension of the cognitive underpinnings of the partial overlap of learning and other developmental disturbances is still lacking. Only a few studies have directly attempted to mark the different and common deficits present in children with dyslexia and dyscalculia. Deficits in phonological skills or in a number module mark the performance of children with dyslexia and dyscalculia, respectively (Landerl et al., 2009; Wilson et al., 2015); in fact, the deficits appear in additive fashion in co-morbid cases. However, this only partially explains the phenomenon because in multiple deficit models (e.g., Pennington, 2006) comorbidity between two conditions is also due to shared etiologic and cognitive risk factors. Although some steps have been taken in the search for the cognitive factors that separately mark the two disorders, identifying the common risk factor seems more difficult. For example, domain general deficits in rapid naming and in verbal short-term memory do not seem to account for the co-morbidity (Wilson et al., 2015). More recently, Slot et al. (2016) have reported that, apart from predicting directly the reading deficit, phonological awareness may represent a risk factor for the co-presence of reading and numerical task deficiencies.

In the present study, we aimed to establish whether performance in reading and numerical tasks can be effectively expressed in terms of global factors. We did not examine whether these factors should be viewed as entirely separate or whether they share some common cognitive elements. In fact, the aims of the present study were mainly descriptive and the present data are not informative on the source of the co-morbidity between dyslexia and dyscalculia. However, we feel that the possibility of identifying the global factors that account for performance in reading and numerical tasks might provide an interesting opportunity for studying the critical factors in determining the two deficits as well as their co-morbidity. In particular, taking into account global components in the data allows controlling for the presence of over-additivity and this could be instrumental in searching for the specific factors underlying a deficit (or the communality between two deficits). In the particular case of dyscalculia, one hypothesis sees the disturbance as due to impaired processing in a number module (Landerl et al., 2004; Butterworth, 2005). Within this perspective, one should find similar deficits for symbolic and non-symbolic stimuli. Testing this hypothesis with reference to global factors can be instrumental as this approach is particularly suited to compare group differences across tasks which may vary considerably for general level of difficulty. Furthermore, other authors have posited that other cognitive factors, such as short-term visuo-spatial memory and inhibition may be critical in explaining the deficient performance in number tasks (Szucs et al., 2013). For example, placing manipulations of these factors within a global component analysis may help understanding whether they add only in terms of task difficulty (which would point to over-additivity) or would produce different selective deficits (which might point to different domains for tasks with high or low visuo-spatial memory components or high and low inhibition components).

One advantage of describing deficits in children with dyslexia and dyscalculia in terms of global models of performance is that it allows determining whether group differences can be ascribed to the decisional or also to the non-decisional part of the response. In particular, the DEM allows isolating a cognitive compartment (marked by the slope of the relationship between condition means and SDs) and a sensory-motor compartment (marked by the intercept of this relationship on the *x*-axis). Previous research has demonstrated that children with dyslexia have a deficit in the decisional compartment of the response but not in sensory-motor compartment (Martelli et al., 2014; for a similar conclusion reached on the basis of the diffusion model see Zeguers et al., 2011). The present results extend this conclusion to numerical tasks. Compared to controls, the children with dyscalculia and those in the mixed pattern group were severely affected on numerical tasks, but (as shown in **Figure 5**) the same relationship between means and SDs held for these groups as well as for the control readers and the children with dyslexia. Therefore, it can be concluded that performance differences among these groups on numerical tasks are actually confined to the decisional component of the response.

Several limitations of the present study must be pointed out. The original sample was moderately large, but some of the target sub-groups were smaller than optimal. Thus, it is possible that the identification of a small group of children with dyslexia may have made it difficult to detect the contribution of specific factors in modulating the deficit (such as the effect of length, which was found in some previous research; e.g., De Luca et al., 2010). At the same time, it should be noted that global components mark quite stable tendencies in the data and, thus, it seems unlikely that part of the present results are unstable. Furthermore, our focus here was on numerical deficits and the sample of children with dyscalculia was comparatively large enough for testing our hypotheses. Nevertheless, confirmation of the present findings in larger subgroups of children is certainly in order. Furthermore, while we tried to have a reasonably comprehensive analysis of reading and number skills, it proved difficult to also examine other co-morbidities, such as ADHD which is well-known to co-occur with learning disorders (Pennington, 2006). ADHD may alter the distribution of RTs (e.g., Hervey et al., 2006). So, it remains a goal for future studies to evaluate whether the co-presence of ADHD symptoms may influence the pattern of findings reported here.

## Conclusion

The present study indicates that the approach of describing the deficit in numerical tasks shown by children with dyscalculia in terms of a global factor is effective, as previously shown in the case of reading deficits of children with dyslexia. As both reading and calculation performance can be effectively expressed in terms of global factors, this perspective may open interesting possibilities to for the study of the frequent, though partial, co-occurrence of these disturbances.

## Author Contributions

All authors listed have made a substantial, direct and intellectual contribution to the work, and approved it for publication.

## Conflict of Interest Statement

The authors declare that the research was conducted in the absence of any commercial or financial relationships that could be construed as a potential conflict of interest.

## References

[B1] Badian N. (1999). Persistent arithmetic, reading, or arithmetic and reading disability. *Ann. Dyslexia* 49 45–70. 10.1007/s11881-999-0019-8

[B2] Barbaresi M. J. Katusic S. K. Colligan R. C. Weaver A. L. Jacobsen S. J. (2005). Math learning disorder: incidence in a population-based birth cohort, 1976–1982, Rochester, Minn. *Ambul. Pediatr.* 5 281–289. 10.1367/A04-209R.1 16167851

[B3] Barbiero C. Lonciari I. Montico M. Monasta L. Penge R. Vio C. (2012). The submerged dyslexia iceberg: how many school children are not diagnosed? Results from an Italian study. *PLOS ONE* 7:e48082. 10.1371/journal.pone.0048082 23118930 PMC3485303

[B4] Barca L. Burani C. Arduino L. S. (2002). Word naming times and psycholinguistic norms for Italian nouns. *Behav. Res. Methods Instrum. Comput.* 34 424–434. 10.3758/BF03195471 12395559

[B5] Biancardi A. Nicoletti C. (2004). *Batteria per la Discalculia Evolutiva (BDE).* Torino: Edizioni Omega.

[B6] Butterworth B. (2005). The development of arithmetical abilities. *J. Chiid Psychol. Psychiatry* 46 3–18. 10.1111/j.1469-7610.2004.00374.x 15660640

[B7] Cerella J. (1985). Information processing rates in the elderly. *Psychol. Bull.* 98 67–83. 10.1037/0033-2909.98.1.67 4034819

[B8] Cornoldi C. Colpo G. (1998). *Prove MT per la Scuola Elementare 2.* Firenze: O.S. Organizzazioni Speciali.

[B9] Cornoldi C. Giofrè D. Orsini A. Pezzuti L. (2014). Differences in the intellectual profile of children with intellectual vs. learning disability. *Res. Dev. Disabil.* 35 2224–2230. 10.1016/j.ridd.2014.05.013 24927516

[B10] Cornoldi C. Lucangeli D. Bellina M. (2002). *AC-MT. Test di Valutazione Delle Abilità di Calcolo.* Trento: Erickson.

[B11] De Luca M. Burani C. Paizi D. Spinelli D. Zoccolotti P. (2010). Letter and letter-string processing in developmental dyslexia. *Cortex* 46 1272–1283. 10.1016/j.cortex.2009.06.007 19631316

[B12] De Luca M. Marinelli C. V. Spinelli D. Zoccolotti P. (2017). Slowing in reading and picture naming: the effects of aging and developmental dyslexia. *Exp. Brain Res.* 235 3093–3109. 10.1007/s00221-017-5041-1 28744622

[B13] de Smedt B. Gilmore C. K. (2011). Defective number module or impaired access? Numerical magnitude processing in first graders with mathematical difficulties. *J. Exp. Child. Psychol.* 108 278–292. 10.1016/j.jecp.2010.09.003 20974477

[B14] Dehaene S. Cohen L. Sigman M. Vinckier F. (2005). The neural code for written words: a proposal. *Trends Cogn. Sci.* 9 335–341. 10.1016/j.tics.2005.05.004 15951224

[B15] Dirks E. Spyer G. van Lieshout E. C. de Sonneville L. (2008). Prevalence of combined reading and arithmetic disabilities. *J. Learn. Disabil.* 41 460–473. 10.1177/0022219408321128 18768777

[B16] Faust M. E. Balota D. A. Spieler H. D. Ferraro F. R. (1999). Individual differences in information-processing rate amount: implications for group differences in response latency. *Psychol. Bull.* 125 777–799. 10.1037/0033-2909.125.6.777 10589302

[B17] Ferraro F. R. (1996). Cognitive slowing in closed-head injury. *Brain Cogn.* 32 429–440. 10.1006/brcg.1996.0075 8975681

[B18] Fias W. Menon V. Szucs D. (2013). Multiple components of developmental dyscalculia. *Trends Neurosci. Educ.* 2 43–47. 10.1016/j.tine.2013.06.006

[B19] Giofrè D. Toffalini E. Altoè G. Cornoldi C. (2017). Intelligence measures as diagnostic tools for children with specific learning disabilities. *Intelligence* 61 140–145. 10.1016/j.intell.2017.01.014

[B20] Hale S. Myerson J. (1996). Experimental evidence for differential slowing in the lexical and nonlexical domains. *Aging Neuropsychol. Cogn.* 3 154–165. 10.1080/13825589608256621 25233058

[B21] Hervey A. S. Epstein J. N. Curry J. F. Tonev S. Eugene Arnold L. Keith Conners C. (2006). Reaction time distribution analysis of neuropsychological performance in an ADHD sample. *Child Neuropsychol.* 12 125–140. 10.1080/09297040500499081 16754533

[B22] Istituto Superiore di Sanità (2011). *Consensus Conference Disturbi Specifici dell’Apprendimento. Sistema Nazionale Per le Linee Guida Ministero Della Salute.* Available https://www.aiditalia.org/Media/Documents/consensus/Cc_Disturbi_Apprendimento.pdf

[B23] Judica A. De Luca M. Spinelli D. Zoccolotti P. (2002). Training of developmental surface dyslexia improves reading performance and shortens eye fixation duration in reading. *Neuropsychol. Rehabil.* 12 177–197. 10.1080/09602010244000002

[B24] Landerl K. Gobel S. Moll K. (2013). Core deficit and individual manifestations of developmental dyscalculia (DD): the role of comorbidity. *Trends Neurosci. Educ.* 2 38–42. 10.1016/j.tine.2013.06.002

[B25] Landerl K. Bevan A. Butterworth B. (2004). Developmental dyscalculia and basic numerical capacities: a study of 8–9-year-old students. *Cognition* 93 99–125. 10.1016/j.cognition.2003.11.004 15147931

[B26] Landerl K. Fussenegger B. Moll K. Willburger E. (2009). Dyslexia and dyscalculia: two learning disorders with different cognitive profiles. *J. Exp. Chiid Psychol.* 103 309–324. 10.1016/j.jecp.2009.03.006 19398112

[B27] Lawrence B. Myerson J. Hale S. (1998). Differential decline of verbal and visuospatial processing speed across the adult life span. *Aging Neuropsychol. Cogn.* 5 129–146. 10.1076/anec.5.2.129.600

[B28] Lewis C. Hitch G. Walker P. (1994). The prevalence of specific arithmetic difficulties and specific reading difficulties in 9- and 10-year-old boys and girls. *J. Child Psychol. Psychiatry* 35 283–292. 10.1111/j.1469-7610.1994.tb01162.x 8188799

[B29] Marconi L. Ott M. Pesenti E. Ratti D. Tavella M. (1993). *Lessico Elementare: Dati Statistici Sull’italiano Scritto e Letto Dai Bambini Delle Elementari [Elementary lexicon: Statistical Data for Italian Written and Spoken by Elementary School Children].* Bologna: Zanichelli.

[B30] Marinelli C. V. Angelelli P. Di Filippo G. Zoccolotti P. (2011). Is developmental dyslexia modality specific? A visual-acoustic comparison on Italian dyslexics. *Neuropsychologia* 49 1718–1729. 10.1016/j.neuropsychologia.2011.02.050 21382386

[B31] Marsh E. B. Hillis A. E. (2005). Cognitive and neural mechanisms underlying reading and naming: evidence from letter-by-letter reading and optic aphasia. *Neurocase* 11 325–337. 10.1080/13554790591006320 16251134

[B32] Martelli M. De Luca M. Lami L. Pizzoli C. Pontillo M. Spinelli D. (2014). Bridging the gap between different measures of the reading speed deficit in developmental dyslexia. *Exp. Brain Res.* 232 237–252. 10.1007/s00221-013-3735-6 24132528

[B33] Mazzocco M. M. Feigenson L. Halberda J. (2011). Impaired acuity of the approximate number system underlies mathematical learning disability (dyscalculia). *Child Dev.* 82 1224–1237. 10.1111/j.1467-8624.2011.01608.x 21679173 PMC4411632

[B34] Myerson J. Hale S. Zheng Y. Jenkins L. Widaman K. F. (2003). The difference engine: a model of diversity in speeded cognition. *Psychon. Bull. Rev.* 10 262–288. 10.3758/BF03196491 12921409

[B35] Myerson J. Lawrence B. Hale S. Jenkins L. Chen J. (1998). General slowing of lexical and nonlexical information processing in dementia of the Alzheimer type. *Aging Neuropsychol. Cogn.* 5 129–146. 10.1076/anec.5.3.182.615 25233058

[B36] Noël M.-P. Rousselle L. (2011). Developmental changes in the profiles of dyscalculia: an explanation based on a double exact-and-approximate number representation model. *Front. Hum. Neurosci.* 5:165. 10.3389/fnhum.2011.00165 22203797 PMC3243900

[B37] Noël M.-P. Rousselle L. De Visscher A. (2016). “Both specific and general cognitive factors account for dyscalculia,” in *Special Needs in Mathematics Education* ed. Lindenskov L. (Copenhagen: Danish School of Education Aarhus University) 35–51.

[B38] Paizi D. De Luca M. Zoccolotti P. Burani C. (2013). A comprehensive evaluation of lexical reading in Italian developmental dyslexics. *J. Res. Read.* 36 303–329. 10.1111/j.1467-9817.2011.01504.x

[B39] Pennington B. F. (2006). From single to multiple deficit models of developmental disorders. *Cognition* 101 385–413. 10.1016/j.cognition.2006.04.008 16844106

[B40] Pennington B. F. Bishop D. V. M. (2009). Relations among speech, language, and reading disorders. *Annu. Rev. Psychol.* 60 283–306. 10.1146/annurev.psych.60.110707.163548 18652545

[B41] Pruneti C. Fenu A. Freschi G. Rota S. Cocci D. Marchionni M. (1996). Aggiornamento della standardizzazione italiana del test delle Matrici Progressive Colorate di Raven (CPM). *Boll. Psicol. Appl.* 217 51–57.

[B42] Rubinsten O. Henik A. (2006). Double dissociation of functions in developmental dyslexia and dyscalculia. *J. Educ. Psychol.* 98 854–867. 10.1037/0022-0663.98.4.854

[B43] Slot E. M. van Viersen S. de Bree E. H. Kroesbergen E. H. (2016). Shared and unique risk factors underlying mathematical disability and reading and spelling disability. *Front. Psychol* 7:803. 10.3389/fpsyg.2016.00803 27375508 PMC4901067

[B44] Szucs D. Devine A. Soltesz F. Nobes A. Gabriel F. (2013). Developmental dyscalculia is related to visuo-spatial memory and inhibition impairment. *Cortex* 49 2674–2688. 10.1016/j.cortex.2013.06.007 23890692 PMC3878850

[B45] Toffalini E. Giofrè D. Cornoldi C. (2017). Strengths and weaknesses in the intellectual profile of different subtypes of specific learning disorder. *Clin. Psychol. Sci.* 5 402–409. 10.1177/2167702616672038

[B46] Tressoldi P. E. Rosati M. Lucangeli D. (2007). Patterns of developmental dyscalculia with or without dyslexia. *Neurocase* 13 217–225. 10.1080/13554790701533746 17999343

[B47] Verhaeghen P. Cerella J. (2002). Aging, executive control, and attention: a review of meta-analyses neurosci. *Biobehav. Rev.* 26 849–857. 10.1016/S0149-7634(02)00071-4 12470697

[B48] Wagenmakers E. Brown S. (2007). On the linear relation between the mean and the standard deviation of a response time distribution. *Psychol. Rev.* 114 830–841. 10.1037/0033-295X.114.3.830 17638508

[B49] Willburger E. Fussenegger B. Moll K. Wood G. Landerl K. (2008). Naming speed in dyslexia and dyscalculia. *Learn. Individ. Differ.* 18 224–236. 10.1016/j.lindif.2008.01.003

[B50] Wilson A. J. Andrewes S. G. Struthers H. Rowe V. M. Bogdanovic R. Waldie K. E. (2015). Dyscalculia and dyslexia in adults: cognitive bases of comorbidity. *Learn. Individ. Differ.* 37 118–132. 10.1016/j.lindif.2014.11.017

[B51] Wilson A. J. Dehaene S. (2007). “Number sense and developmental dyscalculia,” in *Human Behavior, Learning, and the Developing Brain: Atypical Development* eds Coch D. Dawson G. Fischer K. (New York, NY: Guilford) 212–238.

[B52] Zeguers M. H. Snellings P. Tijms J. Weeda W. D. Tamboer P. Bexkens A. (2011). Specifying theories of developmental dyslexia: a diffusion model analysis of word recognition. *Dev. Sci.* 14 1340–1354. 10.1111/j.1467-7687.2011.01091.x 22010894

[B53] Zoccolotti P. De Luca M. Di Filippo G. Judica A. Spinelli D. (2005). *Prova di Lettura di Parole e Non Parole (Words and Non-words Reading Test).* Roma: IRCCS Fondazione Santa Lucia.

[B54] Zoccolotti P. De Luca M. Di Filippo G. Marinelli C. V. Spinelli D. (2017). Reading and lexical-decision tasks generate different patterns of individual variability as a function of condition difficulty. *Psychon. Bull. Rev.* 10.3758/s13423-017-1335-3 [Epub ahead of print]. 28600717

[B55] Zoccolotti P. De Luca M. Di Pace E. Judica A. Orlandi M. Spinelli D. (1999). Markers of developmental surface dyslexia in a language (Italian) with high grapheme-phoneme correspondence. *Appl. Psycholing.* 20 191–216. 10.1017/S0142716499002027

[B56] Zoccolotti P. De Luca M. Judica A. Spinelli D. (2008). Isolating global and specific factors in developmental dyslexia: a study based on the rate and amount model (RAM). *Exp. Brain Res.* 186 551–560. 10.1007/s00221-007-1257-9 18193209

